# Compression of the Ulnar Nerve in Guyon's Canal Caused by a Large Hypothenar Cyst

**Published:** 2009-12-26

**Authors:** Adam Sierakowski, Claire Jane Zweifel, Simon Payne

**Affiliations:** ^a^ St Andrew's Centre for Plastic Surgery and Burns, Broomfield Hospital, Chelmsford, United Kingdom; ^b^ Department of Histopathology, Southend University Hospital, Southend, United Kingdom

## Abstract

**Objective:** We report the case of a 77-year-old man who presented with a long-standing, large swelling of the left hypothenar eminence. This was associated with recent-onset paresthesia and numbness of the ring and little fingers. Magnetic resonance imaging demonstrated a cystic lesion that occupied almost the entire bulk of the hypothenar eminence. **Methods:** Surgical exploration revealed a 7-cm, encapsulated, yellow-brown cyst, around which were stretched the superficial sensory branches of the ulnar nerve. The hypothenar musculature lay flattened against the deep border of the mass. **Results:** The cyst was removed and Guyon's canal was released. Histologic examination confirmed a large cyst containing proteinaceous debris and blood breakdown products. It might have resulted from hemorrhage into a long-standing ganglion. Removal of the cyst led to full resolution of the patient's symptoms. **Conclusions:** This represents an unusual cause of ulnar tunnel syndrome. It is rare to encounter such a large cyst in the hand and interesting in the sense that the resulting symptoms were relatively mild and took many years to develop.

## CASE REPORT

**Figure 1:**
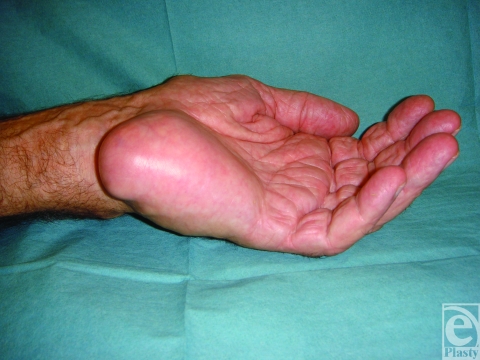
Patient's left hand showing large hypothenar mass.

**Figure 2:**
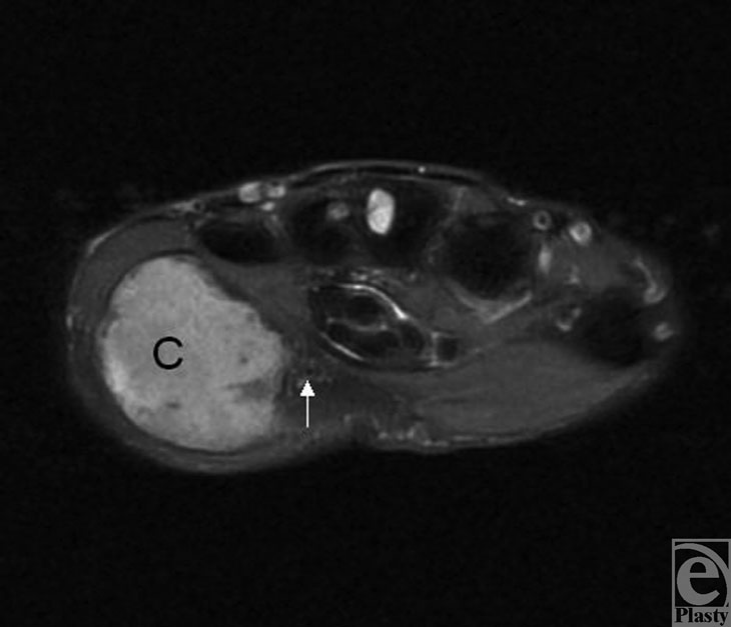
Axial T2-weighted magnetic resonance imaging scan showing loculated lesion (C) of high signal intensity lying in the hypothenar eminence adjacent to Guyon's canal (white arrow).

**Figure 3:**
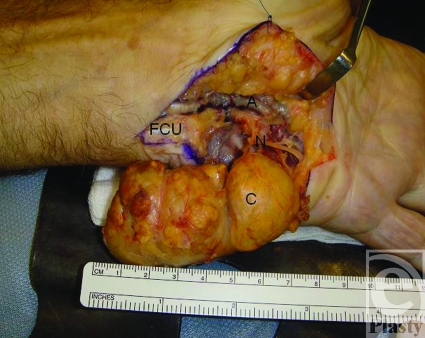
Intraoperative view of the cyst (C) intimately associated with the superficial sensory branches of the ulnar nerve (N). A indicates ulnar artery; FCU, flexor carpi ulnaris tendon inserting onto pisiform.

We report the case of a 77-year-old man who presented with a large swelling of the left hypothenar eminence (Fig 1). According to the patient, a retired fisherman, the swelling had been present for at least 40 years and he had grown accustomed to it. It had only become symptomatic in the last few months, causing paresthesia and numbness of the ring and little fingers. Magnetic resonance imaging demonstrated a loculated mass of 5.8-cm diameter that appeared cystic in nature and occupied almost the entire bulk of the hypothenar eminence (Fig 2). The patient's hand was explored under brachial block anesthesia. Surgery revealed an encapsulated yellow-brown cyst, around which were stretched the superficial sensory branches of the ulnar nerve (Fig 3). The cyst was 7 cm in length, spanning from the distal insertion of the flexor carpi ulnaris tendon to the level of the superficial palmar arch. The hypothenar musculature lay flattened against the deep border of the cyst. The cyst was removed and Guyon's canal was released.

**Figure 4:**
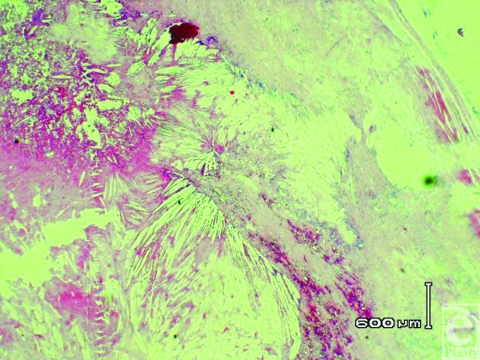
Photomicrograph of the specimen showing fibrotic wall and proteinaceous debris and blood breakdown products with numerous cholesterol clefts inciting a giant cell response (H and E; magnification × 20.)

Histologic examination of the specimen confirmed a large cyst with a thick fibrotic wall. The lumen contained proteinaceous debris and blood breakdown products with numerous cholesterol clefts inciting a giant cell response (Fig 4). In places, the wall was deficient, again replaced by a giant cell response. There was no evidence of malignancy. The cyst did not have specific features to suggest its nature, but hemorrhage into a long-standing ganglion or a host response to implanted squamous material was thought to be the most likely explanation.

## DISCUSSION

After emerging from the forearm, the ulnar nerve enters the wrist passing through Guyon's canal along with the ulnar artery. The canal is approximately 4 cm long, beginning at the proximal extent of the transverse carpal ligament and ending at the aponeurotic arch of the hypothenar muscles. At around the level of the pisiform, the ulnar nerve divides into a deep motor branch and a superficial sensory branch. The deep motor branch, accompanied by the deep branch of the ulnar artery, passes between the abductor digiti minimi and the flexor digiti minimi brevis. It then perforates the opponens digiti minimi and follows the course of the deep palmar arch beneath the flexor tendons. The deep branch of the ulnar nerve provides motor innervation to the muscles of the hypothenar eminence, the interossei, the third and fourth lumbricals, adductor pollicis, and the medial head of flexor pollicis brevis. The superficial sensory branch of the ulnar nerve runs deep and medial to the ulnar artery, lying superficial to the hypothenar fascia. It provides sensory supply to the little finger and the ulnar side of the ring finger.

Any mass growing within or adjacent to Guyon's canal has the potential to cause ulnar nerve compression. Gross and Gelberman^1^ divided Guyon's canal into 3 zones. Ulnar nerve compression within Guyon's canal proximal to its bifurcation (zone 1) results in both motor and sensory deficits. Compression of the motor branch only (zone 2) or the sensory branch only (zone 3) causes purely motor or purely sensory symptoms, respectively. Numerous causes have been reported in the scientific literature, including ganglions, lipomas, anomalous muscle bellies or fibrous bands, ulnar artery disease, and giant cell tumors.^2^^-^5

The cyst described in this case was so large that it spanned all 3 zones and grew within the hypothenar eminence, causing external compression of Guyon's canal and the surrounding structures. What is remarkable is that the patient remained asymptomatic for so long and that when his symptoms did arise, they were limited to sensory disturbance only. It is possible that the deep course of the motor branch of the ulnar nerve spared it from being compressed. At follow-up 4 months postoperatively, the patient's paresthesia had resolved and there were no signs of recurrence. This case demonstrates an unusual cause of ulnar nerve compression in the hand and how even a very large mass of the hypothenar eminence can remain relatively asymptomatic for such a long period of time.
